# Impact of COVID-19 Pandemic on Behavioral and Emotional Aspects and Daily Routines of Arab Israeli Children

**DOI:** 10.3390/ijerph18062946

**Published:** 2021-03-13

**Authors:** Rafat Ghanamah, Hazar Eghbaria-Ghanamah

**Affiliations:** 1 Early Childhood Education Department, Oranim Academic College of Education, Kiryat Tevo’n 3600600, Israel; 2 Edmond. J. Safra Brain Research Center for the Study of Learning Disabilities, University of Haifa, Haifa 3498838, Israel; hazora_7@yahoo.com

**Keywords:** COVID-19, Arab children, lockdown, isolation, behavioral/emotional, daily routines

## Abstract

Negative psychological effects of the coronavirus disease (COVID-19) have been identified in adults and children, such as anxiety and sleep disorders. However, research about the impact of this pandemic on children from ethnical minorities is scarce. We tested the effects of COVID-19 outbreak on psychological aspects and daily routines among Arab Israeli Children. An online cross-sectional survey was conducted among Arab Israeli parents, including behavioral and emotional aspects questionnaire and questions addressing using of screens, sleep, and physical activities. The results showed that, during the COVID-19 outbreak, 55.8% of the children asked to sleep in their parents’ bed and 45% expressed fears they did not have before. Most of the children showed increased irritability, constant mood swings and nervousness about limits and messages, and 41.4% showed sleep difficulties. Concerning adaptive behaviors, more than 50% of the parents reported that their child became wiser, lazier, and was able to adapt the limits and restriction of the COVID-19 outbreak. Moreover, the children tended to increase their use of screens, used to sleep more time, and were less active physically. The results suggest that children are vulnerable to the COVID-19 outbreak psychological effects and highlight the need to reduce the psychological burden of this pandemic and the necessity of immediate intervention.

## 1. Introduction

The outbreak of the Coronavirus disease 2019 (COVID-19) appeared first in China, and then, rapidly, spread to the rest of the world, and the World Health Organization (WHO) declared it as a pandemic [1]. COVID-19 continues to expand and threaten the well-being and the welfare of humans all around the world. Consequently, it has already transmitted to almost 83 million people around the word and led to almost 1.8 million of deaths as of 5 January 2021 [2]. In order to face the COVID-19 spread, all the countries have carried notable efforts addressing the practice of ‘social distancing’. Therefore, many educational institutions, especially schools, have been locked [3,4] and lessons moved to home-based distance-learning models.

Recent researches have proposed that children are not differentiated from older age groups in terms of the risk of infection with the coronavirus; however, they tend to evolve less severe symptoms [5]. The danger of severe disease as well as death is highest for elders and those who suffer from other comorbidities, such as heart illness, cancer, lung disease, and diabetes [5,6]. Nevertheless, children suffer from this pandemic, and they seem vulnerable to its’ severe effects, as they are required to stay home for long periods due to lockdowns and school closing, leading to slight interaction with peers and reduced the chances for exploration and physical activities [6,7,8]. All of these, harmfully, affect children’s welfare, resulting in a various psychological matter, such as anxiety, tension, loneness feeling, depression, sadness, and sleeping problems [7,8,9].

As in the most countries, Israel government forced limitations including physical distancing, and restricted social meetings, gatherings and interactions, sport, public transport, park use, and playground [10]. Israel’s first case of Coronavirus was diagnosed on 26 February 2020. To date (2 January 2021), almost 455,000 people have been infected and 4000 have died from the COVID-19 in Israel. To prevent the outbreak of COVID-19, Israel declared two general lockdowns (March and end of September 2020), and closed the academic institutions. Consequently, most children and youth did not attend schools for long periods, with traditional classroom instructions replaced by learning in capsules (small groups), learning two to three days a week and, especially, online learning activities and home-based learning.

Although the scientific argument is continuous regarding the usefulness of school closings on a virus spread [4,11,12,13], schools have a fundamental role, not just in providing instructive resources to students, but also in offering children the opportunity to connect each other, to communicate with teachers and obtain psychological and emotional counseling [14,15]. Moreover, it has been reported that whenever children are outside of school routines, they are less active physically, spend prolonged time with screens and have unbalanced sleep timetables [7,16,17]. To date, little studies have examined how the quarantine declared due to COVID-19 may affect children’s emotional and behavioral aspects. Some previous studies suggest that the effects may be troublesome [18]. Recent studies found that, compared to pandemic quarantines, being more structured, class days give children more opportunities to be physically active, spend less time in front of screens, and regulate their sleep schedules [7,19]. Additionally, pandemic stressors such as being in isolations, one of the family members infection of Coronavirus, frustration and boredom may have even more negative impacts on children’s behavior and emotions [20,21].

Of relevance for the current study, Arab-Palestinian citizens of Israel (about 1.9 million; 21% of the total number of Israeli citizens) constitute a national and linguistic minority group in Israel [22]. However, in many periods of the pandemic outbreak about 40% of the COVID-19 verified cases involve Arab population, and the positive cases of coronavirus tests reached almost 12% in the Arab population [10]. Experience expanded in prior health crises and from developing reports from the COVID-19 pandemic suggested that ethnic or national minority populations are more vulnerable to the pandemic, as social conditions may worsen the impact of COVID-19 related stressors on overall perceptions of stress and parenting, particularly among minoritized populations [23,24,25]. In addition, given that there are already mental health implications associated with the COVID-19 health crisis, children in families with more risk factors may be more vulnerable to child maltreatment [23]. Arabs in Israel are usually disadvantaged and excluded also in child protective service resources and this situation was worsened during COVID-19 with fewer resources targeted towards them [26]. Thus, it has been expected that the Arabs in Israel, which is demographically considered to be less affluent, having bigger families, higher population density, cultural habits and traditions, living in environmental peripheries, and a superior prevalence of comorbidities, would demonstrate larger rates of infection and more difficult disease course in comparison with the general population [27].

To date, there is little literature available on the impact of COVID-19 pandemic on Arab children’s behavioral, social and emotional aspects, and daily routines. However, a recent study assessed the impact of the COVID-19 outbreak on sleep quality and duration among Israeli children, as reported by Arab and Jewish mothers, showing that the majority of mothers reported no change in their child’s sleep quality and duration [28]. Notably, no differences were found in the study’s variables between Arabic and Jewish participants. Thus, the current study aimed to investigate the impact of the COVID-19 pandemic on behavioral and emotional aspects, and children’s routines activities such as time spent with sleeping and physical activity among Arab children in Israel.

## 2. Materials and Methods

### 2.1. Participants and Setting

The current study was conducted among Arab Israeli parents who have children through an online survey between 4 December to 10 December 2020 after completing long periods of home-quarantine following two general lockdowns and local lockdowns in many Arab areas in Israel. In this study, non-probability sampling (purposive sampling) procedures were used for gathering the primary data from participants. Primarily, Arab parents who had at least one child aged between 5 and 11 years (kindergarten to fourth grade) who were not diagnosed with a physical or psychological problem were selected, a link of designed Google Form of the survey was shared accompanied by an audio clip introducing the research goal on social media including WhatsApp and Facebook, and so on. We focus attention on psychological aspects and daily routines responses of children in these age groups due to the fact that between the age of 4–5 years old vital developmental touch-points have been usually reached (sphincter control, emotional self-regulation, falling asleep in one’s own room, etc.); hence, it is possible to test both regressions in the acquired abilities and the strategies executed by the child to handle with potentially disorganizing conditions [29]. Incomplete forms of the questionnaires and wrong answers such as if the sum of the hours reported exceeded the 24 h or a child reported as infected on COVID-19 but not asked to be in isolation were excluded from the analyses. A total of 390 parents filled the questionnaire and after cleaning the incomplete responses 382 participants were taken for the final analyses.

### 2.2. Data Collection Procedure

Primary data was collected through an online questionnaire since the face-to-face meeting required to be avoided due to ongoing lockdown, and the social distancing still advised. The questionnaire was pilot-tested in a sample of 25 parents before the final study beginning. The questionnaire includes five parts: (i) socio-demographic questionnaire (age, sex, educational level, place of living, any family member of or child was Corona positive or was in quarantine); (ii) regressive behavior of children; (iii) child’s coping behavior against sudden changes in lifestyle; (iv) adaptation behavior of the children; and (v) children’s patterns of use of screens, daily physical activity, and hours of sleep before and during Coronavirus outbreak. Parents were asked to complete the questionnaire (parts ii–v) with reference to two time points: (a) retrospectively 1–2 months before the COVID-19 outbreak in Israel (based on the parents’ memory and knowledge of their children), and (b) during the COVID-19 outbreak. Participants were afforded no financial motivation, and anonymity was maintained to make sure data privacy. Firstly, we asked for the approval of participating in the survey and it was also notified that, at any time, participants could cancel their participation in the survey. This study was carried out online and followed the guidelines of the Declaration of Helsinki and the human research procedure of Oranim College of Education (12/2020-103).

### 2.3. Behavioral and Emotional Measures of Child

An ad-hoc questionnaire was constructed to conduct the survey, included three parts, each part contained four questions, encompassing a total of 12 questions, each question had two possible responses (yes or no). The parts that establish the tool were identified on the base of previous literature on children’s responses to possibly worrying and distressing situations [18,30,31,32,33].

The first area examined the regressive behavior of children with specific focus on the damage of some developmental skills that had been accomplished previously (e.g., sleeping alone, adequacy of language, emotion regulation), (item examples: has your child asked to sleep in his/her parents’ bed in the outbreak period? has your child experienced a general deterioration in their vocabulary across the outbreak period?). The second area studied the child’s opposing behavior against the unexpected change in lifestyle: irritability, continuous mood swings, sleep difficulties, and nervousness about limits and messages coming via media or one of the parents about the COVID-19 (item examples: did your son demonstrate more irritability during the pandemic outbreak period, has your child showed any mood swings that you hadn’t observed before?). Lastly, the third area tested the adaptation behavior of the children with reference to calmness, peacefulness, and adaptation to restrictions, the appearance of listlessness to the activities they were functioning before the COVID-19 outbreak (item examples: during the COVID-19 pandemic, has your child looked calmer and calmer to you? Did your son look wiser and more thoughtful during the COVID-19 pandemic?).

### 2.4. Children’s Patterns of Use of Screens, Daily Physical Activity, and Hours of Sleep before and during Coronavirus Outbreak

Based on prior reports and studies [14,18], the questionnaire included three questions concerning sleeping (how many hours per day did your child sleep before and during the pandemic outbreak?), using of screens (how many hours per day did your child spend in using screen including TV, smart phones and tablets before and during the pandemic outbreak?) and involving in physical activities, i.e., ball games, running, swimming, and walking sports (how many hours per day did your child spend in physical activities before and during the pandemic outbreak?).

### 2.5. Data Analyses

Firstly, descriptive statistics were performed to describe the basic demographic characteristics of the respondents and the children. Secondly, the effects of different variables on the behavioral and emotional aspects were measured by applying chi-square test and the Cramer’s *V* effect size for chi square, the chi-square test was used to measure the association of the variables, with children’s behavioral and emotional aspects.

To allow for comparison between the pre-pandemic and through pandemic routines as number of hours spent in sleeping, physical activity and using screens, a paired sample *t*-test analysis was used.

Additionally, to compare between sub-groups pre and during the pandemic outbreak in terms of hours spent in sleeping, physical activity and using screens, repeated measures analysis of variance (rm-ANOVA) was undertaken. Then, when the rm-ANOVA yielded significant interactions, independent sample *t*-test analysis was applied to find the source of the interactions.

## 3. Results

Table 1 presents the socio-demographic characteristics of the parents and Table 2 presents the socio-demographic characteristics of the children.

Among the participants, there were 329 (86.1%) female and 53 (13.9%) male respondents. Most of the participants tended to be aged 36–45 years (57.9%), total of 68% of the respondents were involved in a job during the lockdown. The respondents were aged 22 to 59 (86.1% were females) (see Table 1 for details).

For children, 51% of the children were males, the most representative grade was the first grade (21.2%), 11.8% of the children were detected in COVID-19 and 43.5% of the children were asked to be in isolation since they had been exposed to COVID-19 diagnosed person (see Table 2).

Reliability test was performed, and the value of Cronbach alpha was high (range between 0.84 and 0.92) showing high level of internal consistency in the three areas investigated.

### 3.1. Regressive Behavior

The analyses showed that, 55.8% of the children, who before the pandemic outbreak used to sleep alone in their own bedroom, asked the parents to sleep in their parents’ bed during the outbreak period. The results showed significant differences between the two pre-school groups indicating that this symptom appeared in the kindergarteners more than in the pre-kindergarten children. In addition, there were significant differences between the different school grades. The follow up analyses showed that this symptom appeared, significantly, in the third grade more than the first and the fourth grade (*χ*^2^ (1, *N* = 105) = 9.16, *p* = 0.002, Cramer’s *V* = 0.30 and *χ*^2^ (1, *N* = 125) = 15.58, *p* < 0.001, Cramer’s *V* = 0.35; respectively) and appeared more in the second grade more than the fourth grade (*χ*^2^ (1, *N* = 127) = 11.32, *p* = 0.001, Cramer’s *V* = 0.30) (see Table 3). Notably, this symptom appeared more in girls than in boys (61.5% vs. 50.3%). The participants who worked during the pandemic reported that their children asked them to sleep beside them more than the participants who did not work (58.7% vs. 45.7%). In addition, analyses showed significant differences between the education groups. The follow up analyses indicated that when the father’s/mother’s education is “MA” degree and over the symptom appeared in their children less than when they have BA degree (*χ*^2^ (1, *N* = 273) = 13.96, *p* < 0.001, Cramer’s *V* = 0.22). Another factor that affects the presence of this symptom, as reported by the participants, is if the child was in isolation, those children asked their parents to sleep beside them more than the children who were not asked to be in isolation. In the families with diagnosed Coronavirus, the children asked to sleep beside the parents more than in families with no diagnosed member (Table 3).

Considering the enuresis, the results indicated that only 5.2% of the children suffered from this symptom. Notably, and despite the fact that this symptom was not common, there were significant differences between the two pre-school groups indicating that this symptom appeared more in the pre-kindergarten children more than in the kindergarten children, in addition, this symptom was greater in the third grade than in the second grade (*χ*^2^ (1, *N* = 114) = 8.91, *p* = 0.003, Cramer’s *V* = 0.28). Remarkably, there were significant difference between girls and boys showing that girls (12%) tended to wet themselves more than boys (0%). However, there were no other significant effects (see Table 3).

Considering the loss of language skills, only 7.3% of the participants reported that their child experienced a general deterioration or loss of their vocabulary. Notably, mothers tended to report that their children showed deterioration in vocabulary during the pandemic more than the fathers. When a member of the family was diagnosed with Coronavirus, parents indicated that their child lost vocabulary more than in families without diagnosed Coronavirus. The analyses showed, also, significant differences between the two pre-school groups indicating that this symptom appeared more in the kindergarten’s children than in the pre-kindergarten children. Moreover, there was a significant effect of the children’s grade. The follow up analyses showed that this symptom appeared, significantly. in the second grade more than in the first and the fourth grades (*χ*^2^ (1, *N* = 105) = 11.42, *p* = 0.001, Cramer’s *V* = 0.33 and *χ*^2^ (1, *N* = 125) = 15.77, *p* < 0.001, Cramer’s *V* = 0.35; respectively) (see Table 3). There was also a significant effect of the gender of the child with boys tended to lose their vocabulary more than girls. Children who were in isolation, significantly, tended to lose vocabulary more than those who were not. Moreover, there was a significant effect concerning the education level of the participants indicating that the parents who have a “BA” degree reported that their children showed a deterioration in their vocabulary more than parents who have a “MA” degree (*χ*^2^ (1, *N* = 273) = 13.41, *p* = 0.011, Cramer’s *V* = 0.16).

With regards to fears, 45% of the participants reported that their child began to express fears they did not have before. Mothers tended to report fears of their children more than fathers during the pandemic. As reported by the parents, significantly, kindergarteners expressed more fear and anxiety than the pre-kindergarteners while there were no significant differences between the elementary school grades (see Table 3). In addition, there was a significant effect of existence of diagnosed Coronavirus in the family with more fear cases reported by parents that a member of the family had been diagnosed with Coronavirus. Parents who worked during the virus outbreak tended to report that their children expressed fears that they did not express before than parents who did not work during the virus outbreak. However, there were no other significant effects.

### 3.2. Opposing Behavior

With regards to irritability, as responded by their parents, 66.2% of the children showed increased irritability. Parents who worked during the outbreak reported existence of this symptom more than parents who did not work, and, when a member within the family was diagnosed with Coronavirus children were more irritable. The results showed that this symptom was more common when the child was in isolation. In addition, this symptom appeared more in kindergarteners than in pre-kindergarteners (see Table 4).

80.9% of the children expressed constant mood swing. There was a significant effect of the parents’ education level. The follow up tests indicated significant differences (*p* = 0.022) between the “BA” degree and the “MA” degree and over (*χ*^2^ (1, *N* = 219) = 7.01, *p* = 0.008, Cramer’s *V* = 0.16). In addition, kindergarteners showed more mood swings than pre-kindergarteners (95% vs. 58%). Moreover, children who were in isolation tended to experience mood swings more than children who were not in isolation. No other significant effects were emerged (Table 4).

Regarding sleep difficulties, 41.4% of the parents reported that their child showed sleep difficulties. The findings showed that this symptom reported more by parents who worked during the Coronavirus outbreak. Remarkably, sleep difficulties appeared more in kindergarteners than in pre-kindergarteners. However, there were no other significant effects (Table 4).

Almost 70% of the parents indicated that their child showed nervousness about the limits and messages coming via media or one of the parents. The results indicated that the pre-kindergarten group showed this symptom less than the kindergarten group). In addition, children who were in isolation showed this symptom more than the children who were not in isolation. The results showed no other significant effects (see Table 4).

### 3.3. Adaptation Behavior

With regards to calmness, children, as reported by the parents, were less calm during the pandemic period (94.8%). The results showed a significant effect of school grade indicating that second graders group reported to be calmer (20.7%) than the first, the third and the fourth grade (0% for the three groups) groups (*χ*^2^ (1, *N* = 107) = 11.42, *p* = 0.001, Cramer’s *V* = 0.33, *χ*^2^ (1, *N* = 114) = 12.95, *p* < 0.001, Cramer’s *V* = 0.34 and *χ*^2^ (1, *N* = 127) = 15.77, *p* < 0.001, Cramer’s *V* = 0.35; respectively). The results showed also that children who were not in isolation reported being calmer than those who were in isolation (see Table 5).

With reference to thoughtfulness, 51% of the parents reported that their child became more thoughtful during the pandemic. Girls were wiser than boys. children who were in isolation had been wiser than those who were not in isolation (59% vs. 44.9%; respectively). Moreover, the pre-kindergarten group were reported as had been wiser than the kindergarten group (see Table 5).

57.6% of the parents responded that their child is lazier during the pandemic than he was before. However, there were no significant effects of any variable (Table 5).

In relation to adaption to the limits and restriction, 66.8% of the participants reported that their children succeeded to adapt the limits and restriction of the COVID-19. The analyses showed a significant effect of parents’ education level indicating that the parents who have a “MA” degree and above reported that their child showed more adaption to restrictions than parents who have a “BA” degree (*χ*^2^ (1, *N* = 273) = 25.59, *p* < 0.001, Cramer’s *V* = 0.31) and parents who have until secondary education (*χ*^2^ (1, *N* = 232) = 8.77, *p* = 0.003, Cramer’s *V* = 0.19). In addition, parents who did not work during the outbreak indicated that their children adapted the limits more than parents who worked through the virus outbreak. Children who were not in isolation tended to adjust to the limits and restrictions more than those who were in isolation. Notably, more adaption to limits reported by parents that none of the family members had been diagnosed with Coronavirus. In addition, a superiority for the girls. There was, moreover, a significant effect of child’s grade in the preschool level and in school grade indicating that the kindergarteners group showed less adaption to the limits and restriction than the per-kindergarteners, and, the fourth grade group outperformed the first and the second grades groups (*χ*^2^ (1, *N* = 118) = 15.11, *p* < 0.001, Cramer’s *V* = 0.36 and *χ*^2^ (1, *N* = 127) = 10.83, *p* = 0.001, Cramer’s *V* = 0.29) and showed more adaption to the restrictions (Table 5).

### 3.4. Children’s Patterns of Use of Screens, Daily Physical Activity, and Hours of Sleep before and during the Quarantine

The analyses for the number of hours spending with screens, physical activities and sleeping, before and during the Coronavirus outbreak (Table 6), indicated significant differences between the two time-points (before and during the outbreak) in spending time with screens with spending more time with screens during the virus outbreak. There were no significant effect of groups or interaction in any variable (see Table 7). Children, as reported by the parents, tend to spend less time in physical activities during the outbreak than before. Notably, the results showed significant interaction of time-point (before and during COVID-19 outbreak) X Diagnosed Coronavirus in the family (positive/negative). Independent sample *t*-test revealed that while there were no significant differences between the two groups (Positive/negative diagnoses in the family) (*t*(380) = 0.49, *p* = 0.625, *d* = 0.05) the analysis showed significant differences during the COVID-19 outbreak (*t*(380) = 2.12, *p* = 0.023, *d* = 0.22), reflecting the fact that when a member of the family diagnosed with Coronavirus the children tended to spend less time in physical activity. However, there were no significant effects of groups or significant interactions concerning the other variables (Table 7). The results showed also significant differences in the number of sleeping hours, with more hours spent in sleep through the outbreak compared to before the outbreak (see Table 6 and Table 7).

## 4. Discussion

This article aims to shed light on the effects the COVID-19 on psychological aspects and daily routines among 5–11 years old Arab Israeli children. The findings will help to inform how best to help and support Arab families with young children through this extraordinary time.

### 4.1. Regressive Behaviors

The results indicate that, during the pandemic, 55.8% of the parents reported that their children asked to sleep beside them, 5.2% reported that their children had enuresis, 7.3% reported language deterioration, and 45% reported an appearance of fears and anxiety. Overall, girls tended to show regressive behaviors more than boys, although boys tended to lose vocabulary more than girls, kindergarteners tended to show regression more than pre-kindergarteners, children who asked to be in isolation and whom a member of the family was diagnosed with coronavirus showed, as reported by the parents, more regressive behavior. In addition, the parents with a “MA” degree or above reported less regressive behavior.

The finding of the current study, especially, regarding fear to sleep alone and the expression of fears and anxiety are in line with recent studies on the effects of COVID-19 outbreak in different countries [6,8,18,33,34], indicating that the Arab children in Israel as other children have shown regressive behaviors. The most common symptoms were the appearance fears and anxiety and asking the parents to sleep beside them. However, Carroll and colleagues [35] who assessed the impact of COVID-19 on health behavior and stress among middle to high income Canadian families with young children found that children seem to be largely protected from fears of COVID-19 as shown by almost half of respondents saying their child experienced ‘very little’ worry and only 7% reported a high concern. It is possible that other stressors such as changes in routines, social isolation from friends, and adjusting to online schooling may be present, but were not captured in their survey.

Remarkably, the two symptoms of asking to sleep beside the parents and the appearance of fears and anxiety, expressed more by children who their parents worked since the pandemic and when one of the family had infected by Coronavirus. Parents who still need to go to the workplace and may lose their jobs tended to increase the behavioral and emotional problems of the children while it decreases for their counterparts [8]. The pressure that caretakers bring home from their workplace can reduce their child-rearing abilities, damage the climate in the home, and in this way bring worry and anxiety into children’s lives. Unfortunately, low-income parents are most apparent to work in stressful, low-quality jobs that prominence low pay and inflexible hours [36].

Social isolation has presented great challenges, for adults, children and adolescents alike. Parents face a frightening prospect of keeping children at home without knowing how long this situation will last [37]. Furthermore, research indicates that the general population, which has symptoms of COVID-19, is more susceptible to stress, anxiety, and depression [38]. While social isolation, by itself, can cause anxiety, depression, stress, and negative feelings [39,40,41].

Notably, our findings are in line with studies with both children and adolescents show that girls are more likely to present anxiety and regressive symptoms [42,43]. 

### 4.2. Opposing Behavior

The finding of the present survey show that, as reported by the parents; 80.9% of the children expressed mood swings, 69.4% expressed nervousness about the messages coming via media or one of the parents, 66.2% of the children showed irritability and 41.4% experienced sleep difficulties, notably, the opposing behaviors, generally, were superior in kindergarten children than in pre-kindergarten children and appeared more when the child was in isolation and when the parents worked during the pandemic.

Expressing irritability and mood swings are common symptoms that reported in many recent studies [7,18,20,38]. Evidence shows that quarantine has adverse psychological effects on adults’ mental health, causing depression, stress, anger, and boredom and that confinement of people at home can produce tensions within households [44,45]. Since that parents in COVID-19 confinement may be particularly worried, these results might reflect less parental emotional accessibility to support children, increasing inadequate parenting practices, such as hostility or inconsistent discipline [46]. Therefore, children’s and adolescents’ symptomatology may rise, as well as the possibility of arguing with family members.

Most of the parents (69.4%) reported that their children expressed nervousness about the messages coming via media or one of the parents. For the general population, among the communication tools, the importance of including codes and available language is emphasized; then, the majority can understand the information presented in the media. In addition, more accurate and relaxing information causing larger satisfaction can result in less stress and depression. News about the number of recovered people, medications, vaccines associated with lower levels of stress [38]. The social media plays an important role in the life of the population in a pandemic context. The effects of messages via media in people’s mental health through the pandemic can contribute to preserving social contact even with physical distance, and also contribute to psychological and medical care through virtual means [47,48]. However, they can also spread fake news and speculation, generating negative feelings [48]. The results show, in addition, children sleep difficulties during the pandemic and indeed, almost 42% of the children had sleep difficulties. This situation can be explained by the anxiety, fears of the virus and the stress period that the children and families face. Stress-related sleep problems are common and particularly during the quarantine period [18,19,33,49,50].

### 4.3. Adaptation Behavior

The findings of the study indicated that, 94.8% of the parents reported that their children were not calmer during the pandemic and 57.6% reported that their children were lazier in this period. However, 66.8% of the participants noticed that their children were able to adjust the limits and restriction of the Coronavirus outbreak. In addition, 51% of the parents declared that their children were more thoughtful during this period. Notably, overall, children who were not in isolation, the fourth-grade pupils and the pre-kindergarten children showed more adaptive behaviors.

Despite the negative effects of the pandemic on adaptive behaviors, e.g., the children were lazier than before pandemic outbreak; however, children as reported by their parents, showed positive adaptive behaviors; they were wiser and were able to cope with the pandemics’ limits. These results may reflect the fact that the pandemic outbreak and the quarantine may lead to positive effects in children [51,52] depending on several variables such as the economic status, social and parental variables [51,53]. As expected, children who were in isolation tended to show lower adaptive behaviors compared to those who were not in isolation. In addition, an advantage for the pre-kindergarteners relative to the kindergarteners was reported. These results are consistent with those found in recent studies concerning the effects of Coronavirus outbreak [54] and specific situation of quarantine during SARS-CoV-2 health crisis on children [6,33]. Despite this coincidence, it is essential to point out that the two previous studies mentioned found a significant increase of symptoms in children aged from 3 to 4 years (pre-kindergarteners) while in our case, these differences were more significant in the kindergarten and elementary school levels. It is possible that the sample size may have influenced these differences.

The difference observed in the present study between kindergarteners and pre-kindergarteners shows that, in some way, pre-kindergarteners are more protected from reality than older children. This protection may derive, on the one hand, from the parents and, on the other hand, from the development and the cognitive systems of young children. Therefore, the care that younger children get from their parents should enable the continuousness of their lives, turning confinement into a time like that of holiday time spent at home or periods of sickness that are so common in young children. It is also likely that these two protecting factors have been less standing in the lives of kindergartens’ and over levels children during quarantine and that part of their emotional and behavioral worsening may reflect the progressive decline in pretend play that is experienced from the ages of 6–7, the more conscious awareness of reality, and the lesser need for constant attention from the parents [54].

### 4.4. Children’s Use of Screens, Daily Physical Activity, and Sleep

The findings of the current study indicate, significantly, changes in the children’s daily routines during the pandemic outbreak and the lockdowns including; increase of daily spending hours in engagement with screen i.e., smartphones, tablets and TV, decrease in physical activity and greater sleeping hours per day. Our results are consistent with many recent researches that demonstrate that when children are out of school, such as during quarantine and holidays, they become physically less active, spend more time facing screens, and face changes in sleep habits [7,16,17,18,19]. During home confinement, children are using screens for more than 4 h, which is certainly higher than levels suggested by international health organizations. For example, the WHO (World Health Organization) suggests limiting screen time to 1 h for children under 6 years, and diverse studies have revealed associations between screen time and lower psychological well-being among children and adolescents. Previous studies have emphasized the costs of over sedentary of the use of screens by children and adolescents on their emotional and behavioral aspects, including lower self-control, fewer emotional stability, more depressive and worry symptoms, and being more tough to deal with [55,56,57]. However, it is important to realize that the present moment is not a holiday, but a health emergency situation that needs the reorganization of activities, which should arrange the organization of days as close as possible to the usual routine, as this can be beneficial against the symptoms of anxiety and stress [58]. Therefore, keeping a daily routine is vital, particularly in homes with the presence of children [39].

Physical activity was reduced significantly in children. Many studies found that the correlation between the reduction of physical activity and psychological well-being is strong and there are evidences that suggest that reduction of physical activity levels may mostly influence mental well-being [59,60,61]. Physical activity, which can be performed at home in the accompanied by parents, who can stimulate their children’s creativity different actions which may contribute to regularization [58]. Notably, in the current study, children in families that a member of them was diagnosed with Coronavirus tend to spend less time in physical activity during the COVID-19 outbreak.

The results of the current study show that children were sleeping more hours in the pandemic period. The results are in line with other studies that showed that during the COVID-19 children increase their sleep hours [7,43,62]. Moore et al. [7] found that children were sleeping more time through a 24-h period than they had been before the COVID-19 pandemic. Likewise, Pietrobelli et al. [62] reported that sleep increased by 0.65 h per day during lockdown among Italian children relative to early 2019. This small increase could be explained by the absence of commuting to school, affording children more time to sleep in later. However, Zreik et al. [28], found that no change in Arab and Jewish Israeli children’s sleep duration during the COVID-19 outbreak compared the period before. This pattern of results may be explained by the age of the children in this study (0.5–6 years old) [28].

For children (5–17-year-olds), 9–11 h of continuous sleep are recommended [63]. Unsatisfactory sleep increases cardiometabolic sickness risk in children and adolescents [64] and leads to anxiety or mood swings, which may be exacerbated by poor mental health during the COVID-19 pandemic [6,38]. With more flexible schedules due to COVID-19-related quarantine measures, children and adolescents may sleep more (closer to achieving recommended guidelines for sleep).

### 4.5. Limitations and Implications for Future Studies

Some limitations of the current study should be mentioned. First, the cross-sectional nature of the study does not allow conclusions on cause–effect relationships, and the enrolment directed through social networks may have caused bias. Second, considering the health threats, a face-to-face interview was avoided while compared to face to-face interviews, an online self-reporting has certain limitations. Finally, it would be better to have a larger sample size to validate the findings but due to the current condition, it was not possible to collect larger samples on a large scale.

Future studies should compare the effects of the COVID-19 between the Arab children and Jewish children in Israel to explore the potential of cultural effects. Further studies should also test possible mediating variables associated with parents or family environment (e.g., parental stress, number of hours working from home) that may reduce parental accessibility and attention as well as parental capacity to manage offspring difficulties and needs.

## 5. Conclusions

We consider that longer periods of social isolation, home confinement, and closure of schools may play a greater risk of psychological distress and increasing of inadvisable habits such as immoderate use of screens. Mental health is a critical concern in a pandemic’s situation and children are considered a susceptible subgroup, thus it is necessitated to reduce the mental health burden of this pandemic among children. Consequently, we suggest that children should preserve a healthy routine with acceptable sleep cycle and physical activity, and media can be used to encourage them to exercise. Parents should permanently talk to their children about the present situations obviously and directly, to minimize the negative feelings and to help the children better understand the pandemic, the information, and messages received from the media. In addition, the government institutions should plan the day next the pandemic by developing intervention and therapeutic programs aiming to contribute to the mental health of children that have been critically diminished, particularly, in national minorities or at-risk populations such as Arab Israeli children.

## Figures and Tables

**Table 1:** Socio-demographic characteristics of parents

Variable	Category	Number	Percentage (%)
Participant’s sex	male	53	13.9
	female	329	86.1
Participant’s age	≤25 years	8	2.1
	26–35 years	113	29.6
	36–45	221	57.9
	46–55	36	9.4
	≥56 years	4	1
Level of education	≤Secondary	109	28.5
	BA degree	150	39.3
	≥MA degree	123	32.2
Are you working right now?	Yes	260	68
	No	122	32
Living area	Urban	132	34.6
	Rural	250	65.4
Are you tensed about your financial condition?	Yes	275	72
	No	107	28
Did one of your family diagnosed with COVID-19	Yes	119	31.2
	No	263	68.8
Did one of your family was on isolation?	Yes	289	75.7
	No	93	24.3

**Table 2:** Socio-demographic characteristics of the children

Variable	Category	Number	Percentage (%)
Gender	Male	195	51
	Female	187	49
Grade	Pre-kindergarten	69	18.1
	Kindergarten	81	21.2
	First grade	49	12.8
	Second grade	58	15.2
	Third grade	56	14.7
	Fourth grade	69	18.1
Diagnosed with COVID-19	Yes	45	11.8
	No	337	88.2
Asked to be in isolation	Yes	166	43.5
	No	216	56.5

**Table 3:** Participants’ answers to the regressive behavior area questions by the study variables

Variable	Category	Question 1 Has Your Child Asked to Sleep in His Parents’ Bed in during the Coronavirus Outbreak?		Question 2 Has Your Son/Daughter Been Wetting the Bed during the Coronavirus Outbreak?		Question 3 During the Coronavirus Outbreak, Has Your Son/Daughter Experienced a General Deterioration in Their Vocabulary in Language Skills?		Question 4 During the Coronavirus Outbreak Has Your Child Expressed Fears that He/She Didn’t Have before?	
		*N* (%)—Yes Answer	*Χ*^2^ (*p*, *V*) Values	*N* (%)—Yes Answer	*Χ*^2^ (*p*, *V*) Values	*N* (%)—Yes Answer	*Χ*^2^ (*p*, *V*) Values	*N* (%)—Yes Answer	*Χ*^2^ (*p*, *V*) Values
Participant’s gender	Male	29 (54.7)	0.03 (0.869, 0.01)	0 (0)	3.40 (0.091, 0.09)	0 (0)	4.87 (0.021, 0.11)	17 (32.1)	4.17 (0.041, 0.10)
	Female	184 (55.9)		20 (6.1)		28 (8.5)		155 (47.1)	
Participant’s education level	≤Sec	60 (55)	13.41 (0.001, 0.19)	5 (4.6)	1.63 (0.443, 0.07)	10 (9.2)	6.45 (0.040, 0.13)	55 (50.5)	1.92 (0.383, 0.07)
	BA	99 (66)		6 (4.2)		15 (10)		63 (42)	
	≥MA	54 (43.9)		9 (7.3)		3 (2.4)		54 (43.9)	
Participant working	Yes	176 (58.7)	4.79 (0.029, 0.11)	14 (4.7)	0.91 (0.340, 0.04)	20 (6.7)	0.91 (0.343, 0.05)	147 (49)	8.92 (0.003, 0.15)
	No	37 (45.1)		6 (7.9)		8 (9.8)		25 (30.5)	
Diagnosed Coronavirus in the family	Yes	83 (69.7)	17.71 (<0.001, 0.19)	4 (3.4)	1.22 (0.269, 06)	20 (16.8)	13.67 (<0.001, 0.19)	66 (55.5)	7.61 (0.006, 0.14)
	No	130 (49.4)		16 (6.1)		0 (0)		106 (40.3)	
Child’s gender	Male	98 (50.3)	4.89 (0.027, 0.11)	0 (0)	22.00 (<0.001, 0.24)	20 (11.4)	5.02 (0.025, 0.12)	97 (49.7)	3.18 (0.074, 0.09)
	Female	115 (61.5)		20 (10.7)		8 (4.5)		75 (40.1)	
Child’s isolation	Yes	110 (66.3)	13.14 (<0.001, 0.19)	8 (4.9)	0.10 (0.749, 0.02)	28 (16.9)	23.22 (<0.001, 0.25)	77 (46.4)	0.22 (0.640, 0.02)
	No	103 (47.6)		12 (5.9)		0 (0)		95 (44)	
Child’s grade	PK	37 (53.6)	17.91 (<0.001, 0.35) 21.81 (<0.001, 0.31)	4 (5.8)	4.82 (0.028, 0.18) 9.31 (0.025, 0.20)	0 (0)	11.11 (0.001, 0.21) 25.93 (<0.001, 0.33)	20 (40.8)	19.81 (<0.001, 0.36) 3.70 (0.296, 0.13)
	K	69 (85.2)		0 (0)		12 (14.8)		53 (65.4)	
	1st	17 (34.7)		4 (8.2)		0 (0)		21 (42.9)	
	2nd	34 (58.7)		0 (0)		12 (20.1)		30 (51.7)	
	3rd	36 (64.2)		8 (16.7)		4 (7.1)		24 (42.9)	
	4th	20 (29)		4 (5.8)		0 (0)		24 (34.8)	

**Table 4:** Participants’ answers to the opposing behavior area questions by the study variables

Variable	Category	Question 1 Did Your Child Show More Irritability (Intolerance to Rules, Caprices, Excessive Demands) during the Coronavirus Outbreak?		Question 2 During the Coronavirus Outbreak, Has Your Son/Daughter Experienced any Mood Swings that You Hadn’t Noticed before?		Question 3 During the Coronavirus Outbreak, Did Your Child Have Sleep Problems that You Had Not Observed before (Difficulty Falling Asleep, Agitation during Sleep, Frequent Awakenings)?		Question 4 Has Your Child Been Nervous about the Pandemic during the Coronavirus Outbreak (When Confronted with Messages Coming from Parents or TV about the Coronavirus; or Because of Restrictions)?	
		*N* (%)—Yes Answer	*Χ*^2^ (*p*, *V*) Values	*N* (%)—Yes Answer	*Χ*^2^ (*p*, *V*) Values	*N* (%)—Yes Answer	*Χ*^2^ (*p*, *V*) Values	*N* (%)—Yes Answer	*Χ*^2^ (*p*, *V*) Values
Participant’s gender	Male	33 (62.3)	0.03 (0.869, 0.03)	49 (92.5)	5.32 (0.021, 0.12)	17 (32.1)	2.19 (0.139, 0.07)	37 (70)	0.01 (0.940, 0.00)
	Female	220 (66.9)		260 (79)		141 (42.9)		228 (69.3)	
Participant’s education level	≤Sec	76 (70)	1.04 (0.596, 0.05)	90 (82.6)	7.48 (0.024, 0.14)	44 (40.4)	0.75 (0.687, 0.04)	79 (72.5)	1.24 (0.538, 0.06)
	BA	99 (66)		129 (86)		66 (44)		105 (70)	
	≥MA	78 (63.4)		90 (73.2)		48 (39)		81 (65.9)	
Participant working	Yes	207 (69)	4.79 (0.029, 0.11)	247 (82.3)	1.88 (0.170, 0.07)	138 (46)	12.40 (<0.001, 0.18)	207 (69)	0.09 (0.763, 0.01)
	No	46 (56.1)		62 (75.6)		20 (24.4)		58 (70.7)	
Diagnosed Coronavirus in the family	Yes	94 (79)	12.59 (<0.001, 0.18)	98 (82.4)	0.24 (0.625, 0.03)	57 (47.9)	3.05 (0.093, 0.09)	90 (75.6)	3.18 (0.093, 0.09)
	No	159 (60.5)		211 (80.2)		101 (38.4)		175 (66.5)	
Child’s gender	Male	126 (64.6)	0.47 (0.496, 0.04)	154 (78)	0.95 (0.331, 0.05)	85 (43.6)	0.82 (0.366, 05)	134 (68.7)	0.08 (0.777, 0.01)
	Female	127 (67.9)		155 (82.9)		73 (39)		131 (70.1)	
Child’s isolation	Yes	133 (80.1)	25.33 (<0.001, 0.26)	145 (87.3)	7.92 (0.005, 0.14)	72 (43.3)	0.49 (0.480, 0.04)	129 (77.7)	9.61 (0.002, 0.16)
	No	120 (55.6)		164 (75.9)		86 (40)		136 (63)	
Child’s grade	PK	40 (58)	5.09 (0.024, 0.18) 2.28 (0.046, 0.03)	40 (58)	29.87 (<0.001, 0.47) 5.57 (0.464, 0.11)	20 (29)	4.41 (0.036, 0.17) 6.83 (0.078, 0.17)	20 (29)	59.11 (<0.001, 0.63) 0.61 (0.894, 0.05)
	K	61 (75.3)		77 (95)		37 (45.7)		73 (90.1)	
	1st	29 (59.2)		41 (83.7)		16 (32.7)		37 (75.5)	
	2nd	34 (58.7)		50 (86.2)		25 (43.1)		42 (75)	
	3rd	36 (64.2)		48 (85.7)		32 (57.1)		40 (71.4)	
	4th	53 (76.8)		53 (76.8)		28 (40.6)		53 (76.8)	

**Table 5:** Participants’ answers to the adaptation behavior area questions by the study variables

Variable	Category	Question 1 During the Coronavirus Outbreak, Has Your Son Seemed Calmer?		Question 2 Did Your Son Seem Wiser and More Thoughtful during the Coronavirus Outbreak?		Question 3 During the Coronavirus Outbreak, Did Your Child Seem Able to Adapt to the Pandemic Restrictions?		Question 4 During the Coronavirus Outbreak, Did Your Child Seem Lazier Than He Was before the Pandemic?	
		*N* (%)—Yes Answer	*χ*^2^/*F* (*p*, *V/η*^2^) Values	*N* (%)—Yes Answer	*χ*^2^/*F* (*p*, *V/η*^2^) Values	*N* (%)—Yes Answer	*χ*^2^/*F* (*p*, *V/η*^2^) Values	*N* (%)—Yes Answer	*χ*^2^/*F* (*p*, *V/η*^2^) Values
Participant’s gender	Male	0 (0)	3.40 (0.091, 0.09)	32 (61.5)	2.14 (0.143, 0.08)	34 (64.2)	1.08 (0.298, 0.05)	36 (68)	0.04 (0.845, 0.01)
	Female	20 (6.1)		163 (50)		186 (56.5)		219 (66.6)	
Participant’s education level	≤Sec	6 (5.5)	3.29 (0.193, 0.09)	54 (49.5)	0.50 (0.779, 0.04)	63 (57.8)	0.10 (0.952, 0.02)	72 (66.1)	25.52 (<0.001, 0.26)
	BA	11 (7.3)		75 (50)		85 (56.7)		81 (54)	
	≥MA	3 (2.4)		66 (53.7)		72 (58.5)		102 (82.9)	
Participant working	Yes	8 (6)	1.65 (0.269, 0.07)	150 (50)	0.61 (0.434, 0.04)	174 (58)	0.10 (0.757, 0.02)	189 (63)	7.72 (0.003, 0.15)
	No	2 (2.4)		45 (54.9)		46 (56.1)		66 (80.5)	
Diagnosed Coronavirus in the family	Yes	6 (5)	0.01 (0.909, 0.01)	66 (55.5)	1.35 (0.246, 0.06)	72 (60.5)	3.05 (0.093, 0.04)	69 (58)	5.99 (0.014, 0.13)
	No	14 (5.3)		129 (49)		148 (56.3)		186 (70.7)	
Child’s gender	Male	9 (4.6)	0.31 (0.578, 0.03)	85 (43.5)	8.87 (0.003, 0.15)	110 (56.4)	0.23 (0.633, 0.02)	118 (60.5)	6.99 (0.008, 0.14)
	Female	11 (5.9)		110 (58.9)		110 (58.8)		137 (73.2)	
Child’s isolation	Yes	3 (1.8)	6.96 (0.008, 0.14)	98 (59)	7.50 (0.006, 0.14)	89 (53.6)	190 (0.168, 0.07)	129 (77.7)	4.61 (0.032, 0.11)
	No	17 (7.8)		97 (44.9)		131 (60.6)		136 (63)	
Child’s age-group (years)	5–6	5 (7.2)	0.93 (0.336, 0.08) 37.96 (<0.001, 0.41)	53 (76.9)	16.18 (<0.001, 0.33) 3.25 (0.354, 0.12)	39 (56.6)	0.00 (0.947, 0.00) 0.69 (0.857, 0.06)	49 (71)	10.71 (0.001, 0.27) 18.04 (<0.001, 0.28)
	6–7	3 (3.7)		36 (44.4)		46 (56.8)		36 (44.4)	
	7–8	0 (0)		25 (51)		27 (55.1)		28 (57.1)	
	8–9	12 (20.7)		29 (50)		35 (60.3)		37 (63.8)	
	9–10	0 (0)		20 (35.7)		31 (55.4)		44 (78.6)	
	10–11	0 (0)		32 (46.4)		42 (60.9)		61 (88.4)	

**Table 6:** Paired sample *t*-test results for the Children’s number of hours a day spent in screens, sleep, and physical activities both before and during the Coronavirus outbreak as reported by their parents

Variable	Before the Outbreak—*M* (*SD*)	During the Outbreak—*M* (*SD*)	*t* (381)	*p* (*d*)
Use of screens	1.75 (0.84)	2.65 (0.89)	17.32	<0.001 (1.77)
Number of sleep hours	8.56 (0.79)	9.17 (0.72)	10.70	<0.001 (1.10)
Physical activity	2.88 (0.92)	2.02 (1.16)	10.74	<0.001 (1.10)

**Table 7:** ANOVA results comparing the time spent in use of screens (number hours a day), sleep and physical activity pre and during the COVID-19 outbreak.

Measure	Variable	Time-Point *F* (*η*^2^)	Group *F* (*η*^2^)	Interaction *F* (*η*^2^)
**Screen time**	Gender	141.02 (0.27) ***	0.10 (0)	0.01 (0)
	Participant’s education level	291.83 (0.44) ***	0.15 (0)	0.01 (0)
	Parents work	199.89 (0.35) ***	0.24 (0)	0.01 (0)
	Diagnosed Coronavirus in the family	249.39 (0.40) ***	0 (0)	0.40 (0)
	Child’s gender	298.99 (0.44) ***	0.89 (0)	0.59 (0)
	Child’s isolation	293.55 (0.44) ***	0.18 (0)	0.02 (0)
	Kindergarten/pre-kindergarten	91.51 (0.38) ***	0.51 (0)	0.01 (0)
	Child’s grade	204.26 (0.47) ***	0.02 (0)	0.12 (0)
**Sleep**	Gender	53.04 (0.12) ***	0.50 (0)	0.02 (0)
	Participant’s education level	113.48 (0.23) ***	0.40 (0)	0.16 (0)
	Parents work	72.97 (0.16) ***	0 (0)	0.16 (0)
	Diagnosed Coronavirus in the family	92.87 (0.20) ***	1.94 (0.01)	0.48 (0)
	Child’s gender	113.90 (0.23) ***	0.31 (0)	1.25 (0)
	Child’s isolation	108.95 (0.22) ***	3.15 (0.01) ^^^	1.81 (0.01)
	Kindergarten/pre-kindergarten	43.96 (0.23) ***	0.37 (0)	0.21 (0)
	Child’s grade	71.96 (0.24) ***	1.26 (0.02)	0.94 (0.01)
**Physical activity**	Gender	64.08 (0.14) ***	0.27 (0)	0.65 (0)
	Participant’s education level	113.52 (0.23) ***	0.24 (0)	0.11 (0)
	Parents work	86.49 (0.19) ***	0.28 (0)	0.72 (0)
	Diagnosed Coronavirus in the family	117.90 (0.24) ***	0.80 (0)	5.18 (0.01) *
	Child’s gender	115.20 (0.23) ***	0.09 (0)	0.17 (0)
	Child’s isolation	117.19 (0.24) ***	0.49 (0)	1.68 (0)
	Kindergarten/pre-kindergarten	54.38 (0.27) ***	0 (0)	0.41 (0)
	Child’s grade	61.13 (0.21) ***	0.24 (0)	1.47 (0.02)

^^^*p* = 0.077, * *p* < 0.05, *** *p* < 0.001. Note*:* Bold type denotes significant statistical difference.

## Data Availability

The data presented in this study are available on request from the corresponding author.
